# Burden of mortality related to sepsis in Brazil from 2002 to 2011

**DOI:** 10.1186/cc12964

**Published:** 2013-11-05

**Authors:** Luciano CP Azevedo, Leandro U Taniguchi, Guilherme PP Schettino, Cristiana M Toscano, Ana L Bierrenbach

**Affiliations:** 1 Instituto Sírio-Libanês de Ensino e Pesquisa, São Paulo, Brazil; 2 State University of São Paulo, Brazil; 3 Federal University of São Paulo, Brazil

## Background

Sepsis represents a substantial healthcare burden. Limited epidemiologic information about the demography of sepsis mortality or about its temporal changes is available. Few population-based sources of data have been used to investigate the burden of sepsis-associated mortality on a national level. We investigated the epidemiology of sepsis deaths in Brazil from 2002 to 2011 using secondary data from the Brazilian Mortality Information System (Sistema de Informações de Mortalidade (SIM)).

## Materials and methods

A retrospective descriptive study using data reported to the Brazilian SIM for the years 2002 to 2011. SIM is an electronic, case-based mortality registry that derives its information almost entirely from death certificates. Sepsis-associated deaths from 2002 to 2011 were identified based on International Classification of Diseases 10th Revision codes listed on the underlying and on the contributing causes-of-death. Population-based sepsis-associated mortality rates and trends were estimated. In addition, age, gender, ethnicity, and outcome variables were assessed. Considering the cases of sepsis identified during the study period, annual population-based mortality rates were calculated using as denominators population estimates provided by the Brazilian Institute of Geography and Statistics with the 2010 census age-stratified population as the standard. Trends of mortality rates over time were explored with the chi-square test for trend. Rate changes were considered significant when *P *< 0.05.

## Results

The total number of deaths recorded in SIM increased over the decade. In 2002 there were 982,294 deaths reported and in 2010 this number was 1,133,761. The number of sepsis deaths increased from 95,972 (9.8%) to 186,712 (16.5%). The average age of sepsis-associated deaths progressively increased from 60.2 years in 2002 to 2003 to 67.1 years in 2010 to 2011. During the same period the average age of all deaths increased from 57.8 years to 62.7 years. White individuals were more frequent (60.4%), as compared with mixed race (24.4%) and blacks (6.6%). A substantial part of sepsis deaths occurred in the hospital (94.8%). The age-adjusted rate of sepsis-associated mortality increased from 69.5 deaths per 100,000 to 97.8 deaths per 100,000 from 2002 to 2010 (*P *< 0.001).

## Conclusions

Between 2002 and 2011, the contribution of sepsis to all-cause mortality increased significantly in Brazil. Moreover, age-adjusted mortality by sepsis also augmented in the last decade. These numbers confirm the importance of sepsis as a significant healthcare issue in Brazil as well as the need for adequate strategies of early recognition and treatment.

